# The rise and spread of invasive emm49 Streptococcus pyogenes in the USA

**DOI:** 10.1099/mgen.0.001615

**Published:** 2026-01-29

**Authors:** Benjamin Metcalf, Srinivas Nanduri, Yuan Li, Zhongya Li, Saundra Mathis, Joy Rivers, Sopio Chochua, Bernard Beall, Lesley McGee, Chris Van Beneden

**Affiliations:** 1 Division of Bacterial Diseases, National Center for Immunization and Respiratory Diseases, Centers for Disease Control and Prevention, Atlanta, GA, USA

**Keywords:** clonal expansion, *emm*49 lineage, group A *Streptococcus*, invasive disease

## Abstract

**Background.** The propensity of *Streptococcus pyogenes* (group A *Streptococcus*) to invade normally sterile sites and cause invasive group A *Streptococcus* (iGAS) disease varies across strains, which are classified using the *emm* gene. Between 2015 and 2017, multistate iGAS surveillance identified an ~150-fold increase of one particular *emm* type, *emm*49. This genomic epidemiological analysis aimed to identify bacterial, patient and societal factors associated with this expansion.

**Methods.** We analysed 1322 *emm*49 iGAS cases and the genome sequences of the clinical isolates acquired through the population-based Active Bacterial Core surveillance during 2015–2022. For each invasive case, we received both a cultured isolate and a standardized case report form that included basic demographic attributes and risk factors of infection. A phylogeographic analysis was performed to reconstruct the divergence times and spatial dispersal history within our *emm*49 collection.

**Results.** Compared to other *emm* types, *emm*49 cases were more common in males (63.5% vs. 58.3%, *P*=0.0143), in people experiencing homelessness (34.0% vs. 17.5%, *P*<0.0001) and in people who inject drugs (23.7% vs. 13.1%, *P*<0.0001). Time-scaled phylogeographic analysis estimated that the most recent common ancestor of the post-2015 expansion isolates occurred around 2004 and that *emm*49 emerged in the western USA.

**Conclusion.** Our findings suggest that the current nationwide outbreak may have originated from the introduction of *emm*49 into disadvantaged (homeless and/or injecting drug users) adult subpopulations. This study underscores how social marginalization and broader social determinants of health can shape iGAS strain epidemiology in the USA.

**Impact Statement**

*Streptococcus pyogenes*, or group A *Streptococcus* (GAS), is a common human pathogen that normally resides in epithelial tissue as non-invasive infections or asymptomatically but is capable of invading normally sterile sites and causing severe invasive group A *Streptococcus* disease (iGAS disease). GAS is commonly characterized by genotyping the highly variable *emm* virulence gene (*emm* typing). As part of ongoing surveillance, the CDC reported a rise in iGAS rates in the USA from 3.6 per 100,000 population in 2013 to 8.2 in 2022. Amid this overall increase, there was a striking increase in one particular *emm* type, *emm*49. Aside from a past outbreak in California, *emm*49 rarely caused invasive disease in Active Bacterial Core surveillance – just 2 cases in 2014. By 2022, it had surged to 297 cases, becoming the most common *emm* type in our surveillance. How did this post-2015 *emm*49 expansion evolve? Was it associated with certain host demographics? In this genomic epidemiological analysis, we find that *emm*49 iGAS was significantly more common in males, persons experiencing homelessness and individuals with an underlying medical history of alcohol abuse. A phylodynamic analysis revealed that the post-2015 expansion originated from a single introduction around 2004 that split into two main clades and that this ancestral strain evolved separately from the earlier *emm*49 California cluster. These findings align with the idea that rising economic and social marginalization, along with social determinants of health, may play significant roles in mediating the evolving epidemiology of invasive GAS strains in the USA.

## Data Summary

All genomic data used in this analysis are available at NCBI under the BioProject accession number PRJNA395240. The authors confirm that all supporting data, code and protocols have been provided within the article or through supplementary data files. Accession numbers of the genomes analysed in this manuscript are listed in Table S1.

## Introduction

*Streptococcus pyogenes* or group A *Streptococcus* (GAS) is a common cause of bacterial pharyngitis and skin infections and can cause serious invasive clinical manifestations including bacteraemia, pneumonia, septic arthritis, necrotizing fasciitis and streptococcal toxic shock syndrome [1]. The propensity to cause GAS invasive group A *Streptococcus* (iGAS) disease varies across strains, which are classified using the *emm* gene that encodes the highly immunogenic M protein [2,3]. Sequence typing of the M protein gene (*emm* typing) has identified over 250 *emm* gene types, with certain types such as *emm*1, *emm*89, *emm*12 and *emm*28 more frequently isolated in past years from invasive and paediatric pharyngitis cases [4]. Long-term surveillance employing *emm* gene typing has been informative in elucidating the ever-changing iGAS strain landscape and plays an important role in understanding the drivers of invasive GAS disease [5,6].

**Fig. 1.**
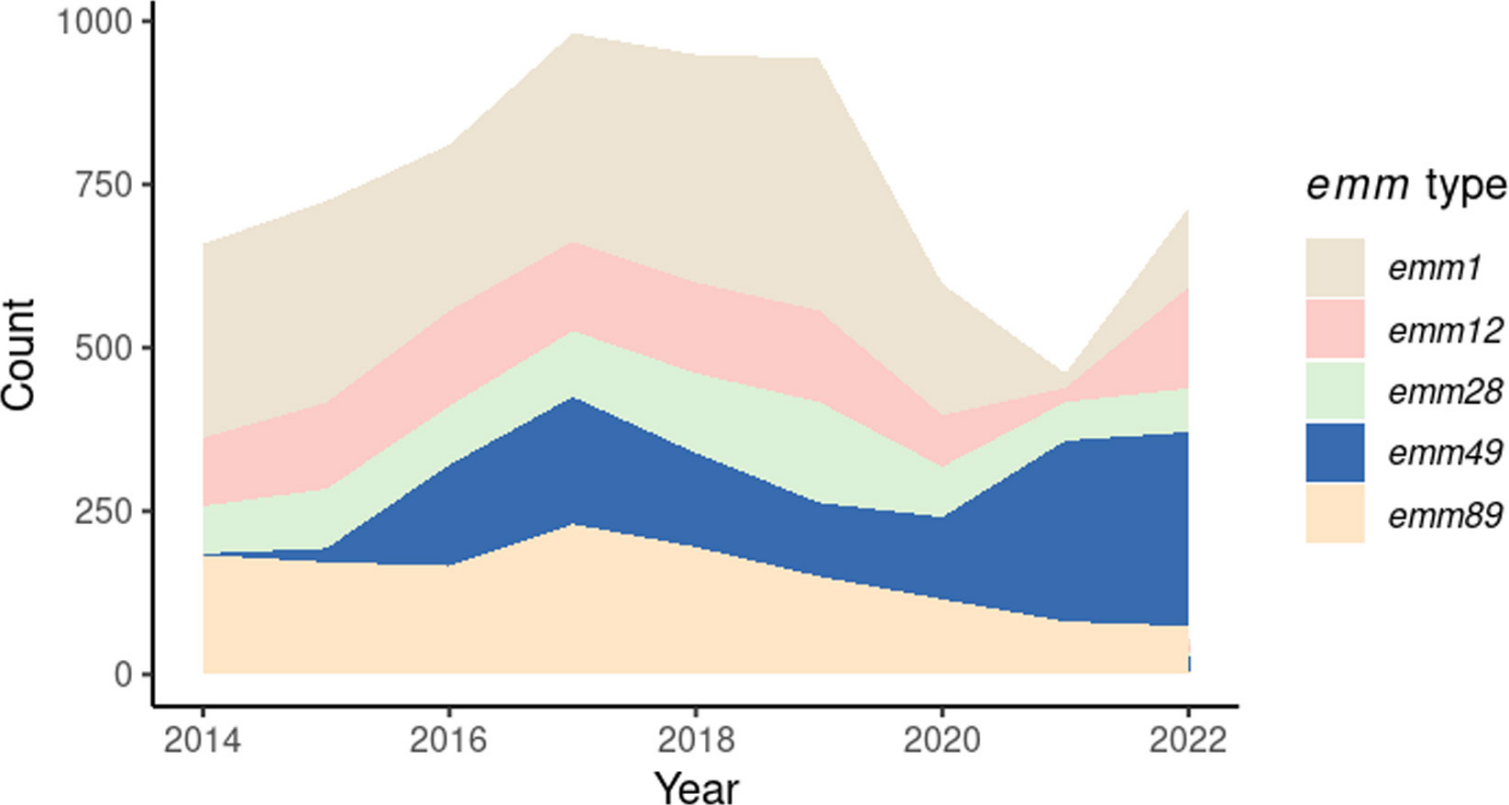
The rapid expansion of ABCs *emm*49 from 2015 to 2022. Plot of *emm*49 iGAS case counts observed through ABCs relative to the top 4 iGAS *emm* types from 2014 reveals the rapid expansion of *emm*49 that began in 2015.

To that end, the Centers for Disease Control and Prevention (CDC) conducts active population- and laboratory-based surveillance for iGAS infections in 10 states in the USA through the Active Bacterial Core surveillance (ABCs) program (https://www.cdc.gov/abcs/bact-facts/data-dashboard.html). Using ABCs, the CDC has found that rates of iGAS disease have increased from 3.6 cases per 100,000 population in 2013 to 8.2 cases per 100,000 population in 2022 in the USA [7]. Within this context of an overall increase, the rapid expansion of one particular *emm* type, *emm*49, has been especially concerning. Besides a brief and localized cluster of cases in California in the early 2000s, *emm*49 rarely caused invasive GAS in the USA. Beginning in 2015, however, *emm*49 iGAS rates began to climb rapidly. As shown in Fig. 1, this previously uncommon type grew from 2 cases (0.2% of 1263 total cases) in 2014 to 195 cases (8.8% of 2206) in 2017, representing the third most common *emm* type observed in our surveillance for that year (https://www.cdc.gov/abcs/bact-facts/data-dashboard.html). In both 2021 and 2022, *emm*49 was the dominant *emm* type observed in ABC surveillance, responsible for 277 (15.2% of 1823) and 297 (12.1% of 2457) cases of iGAS disease, respectively.

What could be causing this post-2015 increase? How did it spread across the USA, and was the expansion associated with certain host demographics? CDC’s ABCs is uniquely qualified to address these questions. For each invasive case in the catchment area, the CDC receives both a cultured isolate and a standardized case report form that includes basic demographic attributes and risk factors of infection. All iGAS isolates are characterized by multiple strain features at the CDC Streptococcus Laboratory, and, since 2015, this characterization has been carried out using whole-genome sequencing. These genomic sequences can also be used in phylogenetic analyses to assess relatedness.

We used ABCs data to address three questions. First, do key patient and demographic characteristics differ between *emm*49 and non-*emm*49 cases? Second, was the recent post-2015 *emm*49 emergence sourced from single or multiple lineages? And third, what is the relationship between the post-2015 emergence of *emm*49 and the cluster of cases in California from the early 2000s? We find that *emm*49 iGAS was significantly associated with several patient features, including being more common in males, persons experiencing homelessness and individuals with an underlying medical history of alcohol abuse. A phylodynamic analysis revealed that the post-2015 expansion originated from a single introduction that split into two main clades and that this ancestral strain evolved separately from the earlier invasive *emm*49 California cluster.

## Methods

### Surveillance

ABCs methods have been detailed elsewhere [4]. Briefly, ABCs sites include San Francisco Bay Area, California (CA) (3 counties); Denver, Colorado (CO) (5 counties); Atlanta, Georgia (GA) (20 counties); Portland, Oregon (OR) (3 counties); Rochester and Albany, New York (NY) (15 counties); select urban counties in Tennessee (TN) (20 counties); Baltimore area, Maryland (MD) (6 counties); and the entire states of Connecticut (CT), Minnesota (MN) and New Mexico (NM), covering a total population of about 34 million persons. ABCs defines an iGAS case as illness in a surveillance area resident with isolation of GAS from a normally sterile site, such as blood or cerebrospinal fluid or isolation of GAS from a wound culture accompanied by a diagnosis of necrotizing fasciitis or streptococcal toxic shock syndrome. Case finding is both active and laboratory-based. To identify cases with GAS sterile site isolates, surveillance personnel reach out to all surveillance area microbiology laboratories and hospitals or obtain electronic line listings where microbiology data are computerized and available. Basic demographics, clinical syndromes and outcomes, underlying conditions and risk factors for infection are extracted from medical charts. Case-patients with GAS cultured from blood, but without a clear clinical syndrome, are categorized as having bacteraemia without a source [8]. Since its inception in 1995, ABCs has collected information on case-patient injection drug use as a risk factor. We classified a case-patient as a person who injects drugs (PWID) if the medical chart indicated injection drug use. Data on whether a case occurred in a person experiencing homelessness (PEH) were collected only beginning in 2010. From 2010 to 2015, ABCs categorized patients as PEH if they were documented in the medical record as homeless or residing in a shelter. Since 2016, ABCs’ definition of homeless has been expanded to include patients who resided in a mission, medical respite or church community centre at the time of positive culture [9].

### Laboratory methods

All iGAS isolates collected from ABCs cases are characterized by the CDC’s Streptococcus Laboratory for multiple strain features, including the *emm* type. Prior to 2015, the isolate *emm* type was determined by sequencing *emm-*specific PCR amplicons. Since 2015, all isolates undergo whole-genome sequencing (WGS), and their *emm* type is determined from the WGS data as described [10]. All available pre-2015 *emm*49 isolates also underwent sequencing. All genomic data used in this analysis are available at NCBI under the BioProject accession number PRJNA395240.

### Bioinformatic analysis

We use a validated bioinformatics pipeline available at https://github.com/BenJamesMetcalf for determining *emm* types, multi-locus sequence types, resistance determinants and other virulence genes from the available sequence data [11].

#### Core SNP phylogenetic comparisons of 1322 *emm*49 and 62 *emm*151 genomes

A phylogeny of 726 *emm*49 isolates representing cases from 1998 through 2019 was reconstructed using the kSNP v3.0 software [12]. Specifically, we used a core SNP alignment generated using the ‘core’ argument and a kmer length of 19 to build a parsimony tree. Inclusion criteria for phylogenetic analysis required genome assemblies to have ≤200 contigs and an N50 ≥30000. The tree was visualized and annotated in iTOL v.3.2.4 [13]. Following the same methodology, a second phylogeny of 596 *emm*49 and 62 *emm*151 isolates acquired through ABC surveillance from 2020 through 2022 was also produced.

#### Time-scaled *emm*49 phylogeography

We generated a time-measured phylogeny using BEAST v.2.5.2 after assessing the strength of the temporal signal of the collection using TempEst v1.5.3 employing the heuristic-residual-mean-squared method to find the best-fit root [14,15]. Signal of individual subclades on the phylogeny was estimated with Clockor2 v.1.9.1 using the residual-mean-squared method to find the best-fit root [16]. The phylogeny produced by TempEst was used as the starting tree for the BEAST analysis. The time-based phylogeographic tree was reconstructed with an HKY substitution model, a Gamma site heterogeneity model, a strict molecular clock [with a Gamma (*α*=0.001, *β*=1,000) ‘clock.rate’ prior] and a constant coalescent tree prior. We used a discrete phylogeographic approach using a continuous-time Markov chain, where each U.S. state in ABCs surveillance was treated as a unique site. We used the core SNP alignment generated by kSNP3. Ascertainment bias correction was carried out with the following estimates of invariant sites: A=573,536, C=357,043, G=360,781, and T=574,702.

We used Tracer v.1.7.1 to assess the Markov chain Monte Carlo chain length with a 10% burn-in needed to achieve an effective sample size >200 for all traces [17]. TreeAnnotator v.2.5.2 was used to generate a maximum clade credibility tree with a 10% burn-in and median node heights. The time-scaled tree was visualized and annotated using FigTree v.1.4.2. Finally, to visualize the spatiotemporal dispersal history of *emm*49 embedded within the phylogeographic reconstruction, we used SpreaD3 v.0.9.7.1 [18]. All the in-house scripts and XML files used in this analysis are available at https://github.com/BenJamesMetcalf/emm49_US_analysis.

### Statistical analysis

We compared demographic and clinical characteristics and risk factors among *emm*49 and non-*emm*49 case-patients for the years 2015–2019 from the four ABCs sites responsible for the largest proportion of *emm*49 cases during this period. We compared categorical variables between groups using the chi-square and Fisher’s exact tests, where one of the cells contained a value of less than 5. *P* values <0.05 were considered statistically significant.

## Results

**Fig. 2.**
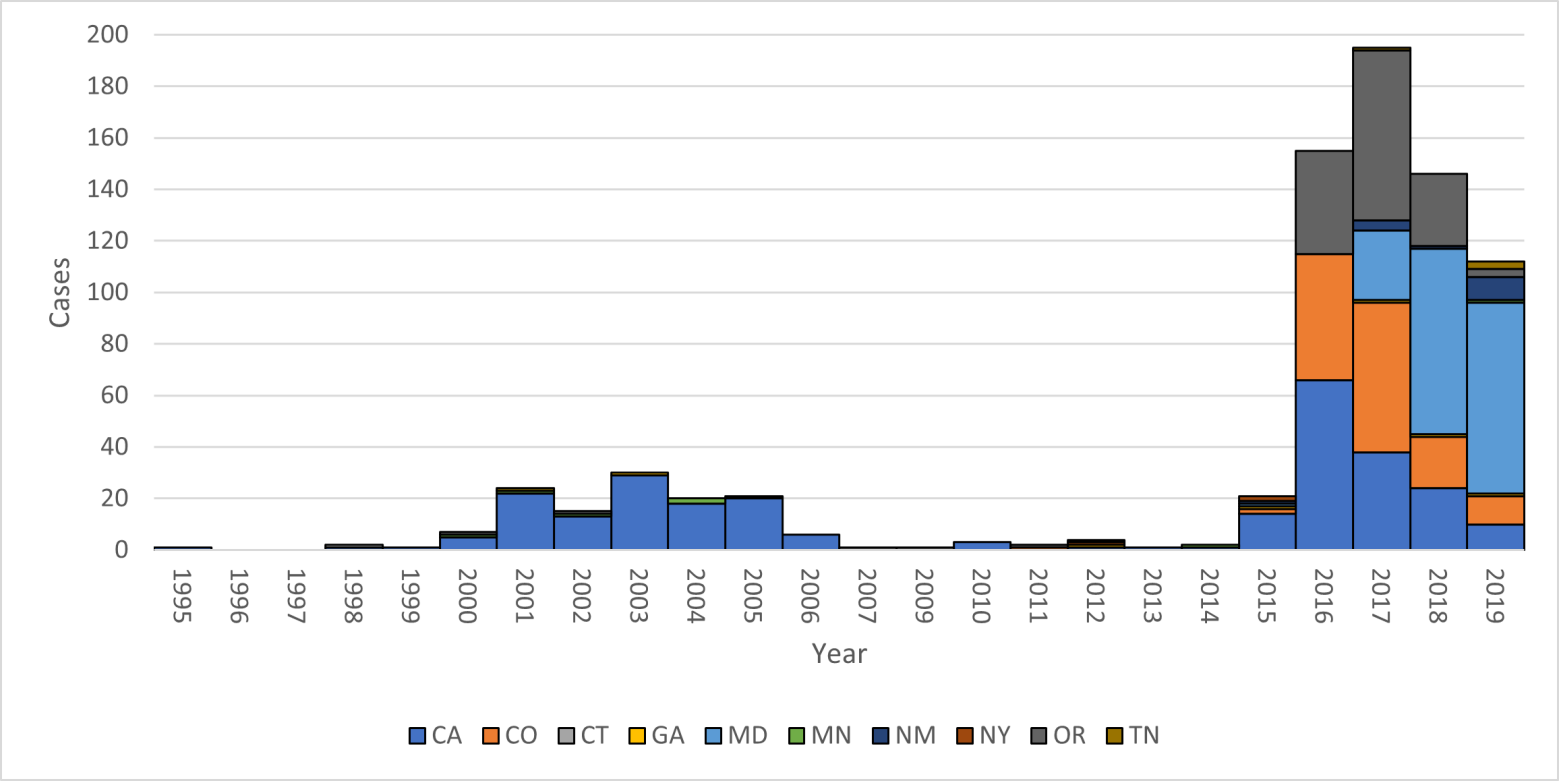
*emm*49 invasive GAS cases reported by ten participating sites in Active Bacterial Core surveillance, 1995–2019

From 1995 to 2019, ABCs identified 770 *emm*49 iGAS cases (2.9% of 26,223 total cases). Annual counts of *emm*49 iGAS cases remained below 5 (range 0–4) per year before 2015 (average 0.18% of total cases per year), except for a 7-year period from 2000 to 2006 when there were 123 cases identified, averaging 18 cases per year (range 6–30; 2.0% of total cases per year) with 113 (91.9%) from the CA site alone. The number of *emm*49 iGAS cases increased beginning in 2015 with 21 identified in 2015, 155 in 2016, 186 in 2017, 145 in 2018 and 112 in 2019 (Fig. 2). Of 629 *emm*49 invasive GAS cases identified in 2015–2019 (average 6.3% of total cases per year), 603 (95.9%) were from 4 sites: CA, CO, OR and MD [Figs S2–S5 (available in the online Supplementary Material) for epi curves by site for 2015–2019]. Table 1 provides descriptive epidemiology of these invasive *emm*49 cases compared to cases caused by other *emm* types from these four sites.

**Table 1.** Comparison of characteristics of persons with *emm*49 cases and non-*emm*49 cases, four ABCs sites (CA, CO, OR and MD), 2015–2019

	*emm*49 no. (%)	Non*-emm*49 no. (%)	*P* value*
Total iGAS cases	603	4,286	
Male	383 (63.5)	2,497 (58.3)	**0.0143**
*Age group in years*			
<18	7 (1.2)	249 (5.8)	**<0.0001**
18–34	113 (18.7)	662 (15.5)	**0.0381**
35–49	145 (24.1)	871 (20.3)	**0.0348**
50–64	229 (38.0)	1,193 (27.8)	**<0.0001**
65 and older	109 (18.1)	1,311 (30.6)	**<0.0001**
*Outcome*			
ICU admission	221 (36.7)	1,671 (39.0)	0.2699
Death	38 (6.3)	364 (8.5)	0.0667
*Clinical syndrome*‡			
Cellulitis	271 (44.9)	1,894 (44.2)	0.7279
Bacteremia without focus	167 (27.7)	914 (21.3)	**0.0004**
Septic shock	80 (13.3)	753 (17.6)	**0.0085**
Pneumonia	62 (10.3%)	550 (12.8)	0.0764
Abscess	48 (8.0)	337 (7.9)	0.9337
Osteomyelitis	24 (4.0)	257 (6.0)	**0.0464**
Necrotizing fasciitis	24 (4.0)	210 (4.9)	0.3220
Endocarditis	14 (2.3)	83 (1.9)	0.5254
Meningitis	4 (0.7)	30 (0.7)	0.2075†
Streptococcal toxic shock syndrome	2 (0.3)	72 (1.7)	**0.0030†**
*Underlying conditions or risk factors*$			
History of penetrating or blunt trauma	134 (22.2)	948 (22.1)	0.9542
Diabetes	116 (19.2)	1,117 (26.1)	**0.0003**
Alcohol abuse, current	107 (17.7)	485 (11.3)	**<0.0001**
Cirrhosis	90 (14.9)	302 (7.1)	**<0.0001**
Chronic skin breakdown	82 (13.6)	517 (12.1)	0.2814
Obesity	76 (12.6)	671 (15.7)	0.0511
Chronic kidney disease	66 (11.0)	651 (15.2)	**0.0058**
HIV	32 (5.3)	108 (2.5)	**0.0001**
*Risk groups*			
PEH	205 (34.0)	750 (17.5)	**<0.0001**
PWID	143 (23.7)	561 (13.1)	**<0.0001**
Both PEH and PWID	77 (12.8)	264 (6.2)	**<0.0001**
**Either PEH or PWID**	271 (44.9)	1,047 (24.4)	**<0.0001**

*P-values were generated using the chi-square test to compare categorical variables between groups.

†*P*-value from the Fisher’s exact test

‡Some cases had more than one syndrome identified; no other syndrome overlapped with ‘bacteraemia without focus’.

$Some cases had more than one underlying condition or risk factor.

### Descriptive epidemiology of *emm*49 vs. non-*emm*49 iGAS cases identified from CA, CO, OR and MD in 2015–2019

Of the overall 4,889 iGAS cases identified from CA, CO, OR and MD sites in 2015–2019, there were 603 (12.3%) *emm*49 iGAS cases. Cases due to *emm*49 were more frequent in males when compared to non-*emm*49 iGAS cases (63.5% vs. 58.3%, *P*=0.0143). The proportion of *emm49* iGAS case-patients who were 18–34 years of age, 35–49 years of age or 50–64 years of age was significantly higher compared to the proportions seen in non-*emm*49 cases, and the proportion of *emm*49 iGAS patients who were <18 years or ≥65 years was significantly lower than the proportion seen in non-*emm*49 iGAS case-patients (Table 1). Overall, the proportion of *emm*49 cases that were 18–64 years of age was higher than the proportion of non-*emm*49 cases that were in this age group (80.8% vs. 63.6%, *P*<0.0001). When compared with non-*emm*49 cases, the proportion of cases presenting with bacteraemia without focus was significantly higher among *emm*49 cases (21.3% vs. 27.7%, *P*=0.0004), and the proportion of cases presenting with septic shock (17.6% vs. 13.3%, *P*=0.0085), streptococcal toxic shock syndrome (1.7% vs. 0.3%, *P*=0.0030) or osteomyelitis (6.0% vs. 4.0%, *P*=0.0464) was significantly lower among the *emm*49 cases (Table 1). When compared with non-*emm*49 cases, a significantly higher proportion of *emm*49 cases were identified with an underlying medical history of current alcohol abuse (11.3% vs. 17.7%, *P*<0.0001), human immunodeficiency virus (HIV) (2.5% vs. 5.3*%, P*=0.0001) and cirrhosis (7.1% vs. 14.9%, *P*<0.0001).

Of the 603 *emm*49 cases, 205 (34.0%) were identified as PEH and 143 (23.7%) as PWID from medical record review. There were 77 (12.8%) *emm*49 cases who were identified as both PEH and PWID. Nearly half of the *emm*49 cases were either PWID or PEH (44.9%); this was 1.8 times (95% confidence interval (CI) 1.6, 2.1) the proportion of PWID or PEH among non-*emm*49 cases (24.4%) (Table 1). The proportion of *emm*49 iGAS cases identified as either PEH or PWID varied by site: 48.6% (74/152) in CA, 39.3% (55/140) in CO, 48.2% (66/137) in OR and 43.7% (76/174) in MD.

### *emm*49 phylogenetic analysis

**Fig. 3.**
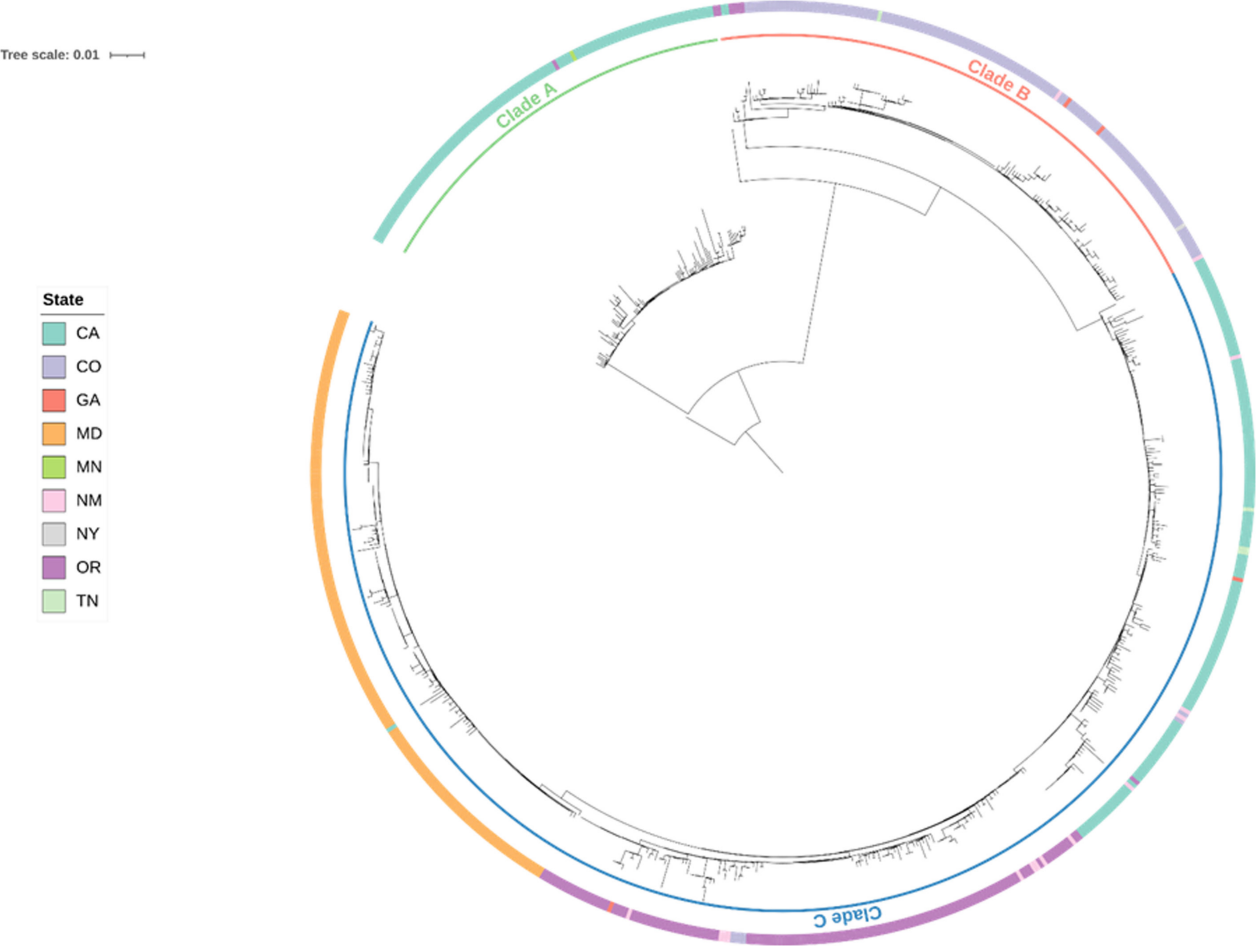
A midpoint rooted phylogeny representing 726 *emm*49 isolates constructed from an alignment of 782 core SNPs. Phylogeny shows that *emm*49 invasive isolates were comprised of three major clades (designated as clades A, B and C) collected across nine ABCs sites. The ABCs collection site for each patient case is annotated on the tree using the outer colour strip. Clade A is the oldest clade, with isolate culture dates ranging from 12 April 2000 to 27 July 2006, while clades B and C are more recent, with culture dates spanning 21 May 2012 to 30 December 2019.

We generated a core SNP phylogeny to assess the relatedness among 726 *emm*49 isolates acquired through ABC surveillance from 1998 through 2019 after removing an outlier group of 22 isolates. The tree indicated that invasive *emm*49 genomes were structured around three major clades, which we designated as clade A, clade B and clade C (Fig. 3). Clade A is composed of 106 genomes and represented the oldest collected surveillance cases, with culture dates ranging from 12 April 2000 to 27 July 2006, and an average pairwise SNP distance of 4.84 (range: 0–21). Clade B consisted of 147 genomes and represented a more recent collection of cases occurring from 21 May 2012 to 28 December 2019, with an average pairwise distance of 3.95 SNPs (range: 0–16). Lastly, clade C was the largest clade, consisting of 471 case isolates gathered during a similar timeframe as clade B (9 September 2014 to 30 December 2019), with an average pairwise distance of 6.64 SNPs (range: 0–23). By comparing the phylogenetic distance from the most common recent ancestor (MRCA), we observed a notable amount of genomic distance between the oldest clade A and clades B and C (~86 SNPs) relative to the distance between the more recent clades B and C (~18 SNPs).

At least one *emm*49 isolate was collected from nine of the ten ABCs sites, excluding CT. Each clade was represented in multiple geographical locations, although a particular ABCs site (or sites) dominated each (Fig. 3). Clade A isolates were primarily from CA [104 of 106 (98.1%) isolates] but also included one isolate each from MN and OR. Clade B was predominantly acquired from CO [134 of 147 (91.8%) isolates] but also included isolates from CA [2], NM [2], NY [1], OR [5], GA [2] and TN [1]. Clade C had a substantial presence in CA [151 of 471 (32.1%) isolates], OR [133 of 471 (28.2%) isolates] and MD [163 of 471 (34.6%) isolates] and also included isolates from CO [6], GA [2], NM [13] and TN [3]. The results indicate that both clade A and clade B were fairly cloistered, with neither expanding much beyond their emergent sites (CA and CO, respectively), while clade C had a strong presence in multiple locations and drove a greater share of the recent spread of *emm*49 after 2015.

PEH and/or PWID represented a large proportion of case-patients contributing isolates across all three clades, including 26% (27 of 106 case-patients) of clade A, 40% (58 of 134 case-patients) of clade B and 45% (213 of 471 case-patients) of clade C (Fig. 3). The lower percentage observed for clade A reflects only PWID cases, as data on PEH status was not included in case-patient metadata until 2010, by which time clade A was no longer circulating.

### *emm*49 Antimicrobial Resistance

Among the 770 *emm*49 isolates gathered in this collection, 2 levofloxacin non-susceptible isolates, 123 tetracycline-resistant isolates (16%) and 118 erythromycin- and clindamycin-resistant (117 were *ermTR-*positive and 1 *ermB-*positive, associated with clindamycin inducible and constitutive phenotype, respectively [19]) isolates (15.3%) were identified. The proportion of combined erythromycin (ERY) and clindamycin (CLI) resistance (15.3% vs. 21.5%, *P*<0.001), as well as tetracycline resistance (16% vs. 26.2%, *P*<0.001), was less common in *emm*49 when compared to non-*emm*49 iGAS isolates. A unique ERY and CLI resistance profile was observed within clade A and within a subclade from clade C (Fig. S1). Within clade A, 68 isolates (64% of clade A) were ERY- and CLI-resistant. Clade C was mostly ERY- and CLI-sensitive, but a subclade of 38 isolates (8% of clade C) from MD acquired resistance to ERY and CLI. This resistant subclade grew rapidly and appeared to replace susceptible *emm*49 within the state; however, it is unknown whether the resistance offered any selective advantage in its expansion since clade C overall was rapidly increasing during this timeframe. Additionally, there were six sporadic isolates, three from clade B (2% of clade B) and four from clade C, which were *ermTR-*positive.

### Time-scaled phylogeographic analysis

**Fig. 4.**
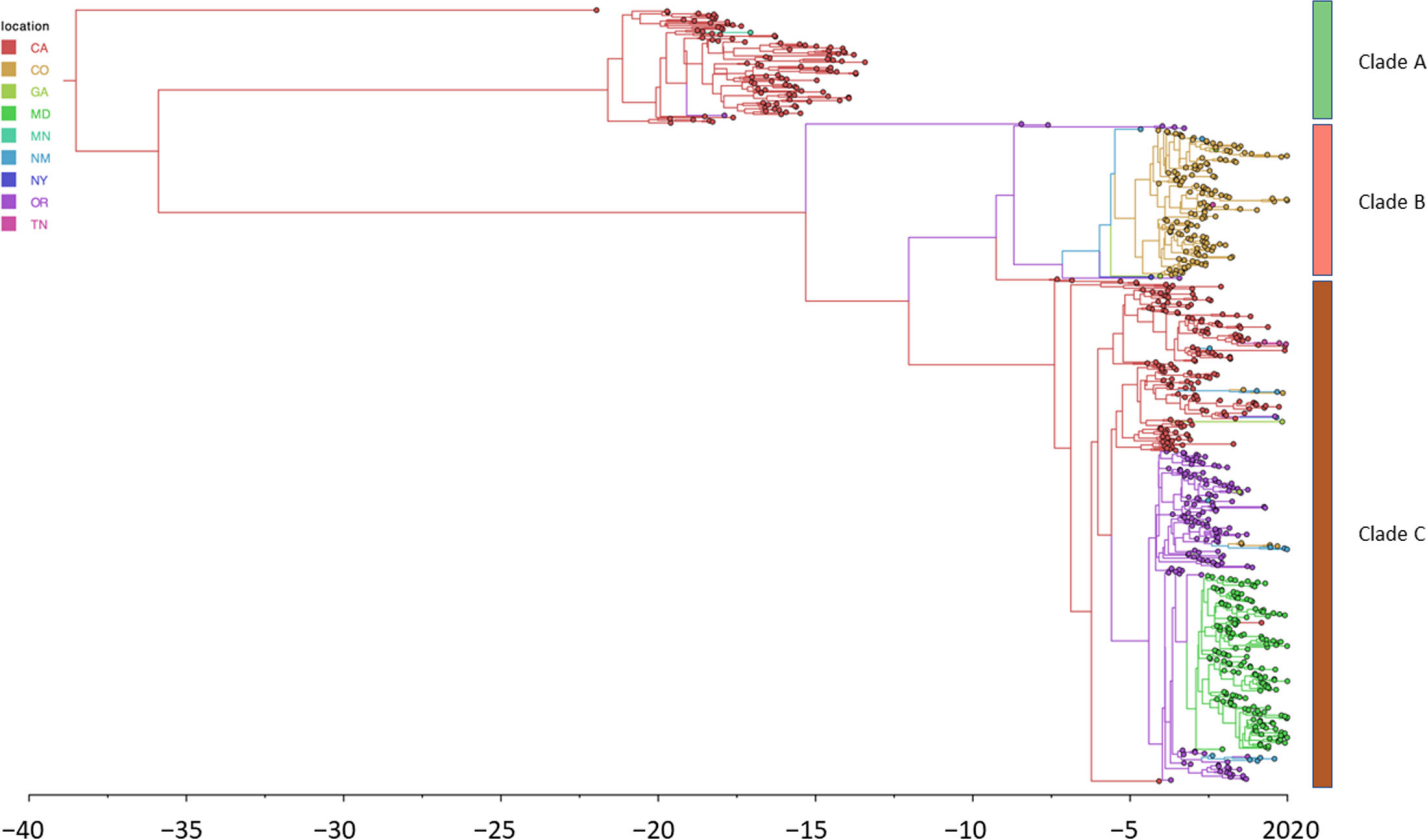
Time-measured phylogeography of circulating invasive *emm*49 strains suggests recent, and possibly multiple, introductions into the USA. The tMRCA of the three clades (designated by vertical colour bars) representing the entire collection of 726 isolates was estimated to be 23 April 1981 (range: 10 February 1976–19 April 1986; 95% HPD). The oldest clade, clade A, emerged about 17 years later, 26 April 1998 (range: 21 February 1997–28 May 1999; 95% HPD). Both clade B and clade C appeared later, approximately 8 September 2010 (range: 31 July 2009–20 September 2011; 95% HPD) and 13 July 2012 (range: 5 April 2011–15 August 2013; 95% HPD), respectively.

To track the evolution of the tip-dated samples over the course of the study, we generated a phylogeographic reconstruction using the BEAST2 software. As a preliminary step, we assessed the strength of the temporal signal in the dataset by calculating the correlation between the divergence from the root of the tree and sampling time using the TempEst and Clockor2 programs. A correlation of 0.93 for the overall collection as well as correlations of 0.40, 0.43 and 0.48 for clades A, B and C, respectively, were judged suitable for moving forward with the reconstruction (Fig. S6). Time-scaled phylogenetic analysis found that the time to most recent common ancestor (tMRCA) of the 726 isolates comprising the entire collection was around April 1981 [95% highest posterior density (HPD): 10 February 1976–19 April 1986] (Fig. 4). The tMRCA of clade A was estimated to be in April 1998 (95% HPD: 21 February 1997–28 May 1999), clade B arrived later, approximately September 2010 (95% HPD: 31 July 2009–20 September 2011), and clade C appeared most recently, around July 2012 (95% HPD: 5 April 2011–15 August 2013). The phylogeography suggests that clade B was causing sporadic disease initially in OR and CA and then NM before establishing an outbreak in CO. Clade C initially established a foothold in CA and then expanded into OR followed by MD.

As shown in Fig. 2, *emm*49 invasive disease within ABCs can be broadly categorized into two expansions, with the first represented by clade A (2000–2006) and the second represented by clades B and C (2015–2020). To understand how these two expansions are related to each other, we examined three possible scenarios. In the first, clade A (expansion 1) is a direct ancestor of clades B and C (expansion 2). In the second, the strain that gave rise to lineages B and C did not evolve directly from clade A but emerged in the population at the same time as clade A. This would be the case if there were a single introduction of two strains, one that gave rise to clade A and another that later gave rise to clades B and C. In the last scenario, the first expansion (clade A) and the second expansion (clades B and C) represent separate introductions of independent strains at different times. The time-measured phylogeny in Fig. 4 enabled us to assess the evidence for each scenario presented. We did not see support for scenario 1 since the monophyletic group that included all clade A isolates did not include the MRCA of the clades B and C isolates, indicating that clades B and C did not evolve directly from clade A (Fig. 4). Phylogenetic analysis also did not support scenario 2, which suggested that the isolates from clade A and the strain that gave rise to clades B and C emerged at the same time. The time-measured phylogeny estimated that the tMRCA of clades B and C was around November 2007 (range: 5 March 2006–17 August 2009; 95% HPD), which was after the emergence of clade A. Finally, if scenario 3 was correct, we would expect a large amount of divergence between clade A and the strain that gave rise to clades B and C. We would also expect the tMRCA of all three clades to be much earlier than the first cases of clades A, B or C. We observed phylogenetic support for this possibility, with the tMRCA of clades A, B and C estimated to be in January 1984 (range: 24 May 1979–28 September 1988; 95% HPD) (Fig. 4). This tMRCA existed ~16.3 years before the first cases of clade A and 28.4 years before the first cases of clades B and C. Thus, the evidence suggests that the strains that gave rise to the first and second expansions may represent separate introductions at separate times.

### *emm*49 invasive disease since 2020

The eastward expansion of *emm*49 invasive disease across the USA preceded the coronavirus disease 2019 (COVID-19) pandemic, and *emm*49 was already well established before any non-pharmaceutical interventions were enacted. For invasive GAS overall, work by Prasad *et al.* showed that the onset of social distancing measures correlated with lower disease rates [20]. Using seasonal autoregressive integrated moving average models, they found that observed iGAS incidence was 28% lower than expected [20]. However, this trend did not seem to hold for *emm*49, with cases increasing from 112 in 2019 to 297 in 2022 (https://www.cdc.gov/abcs/bact-facts/data-dashboard.html). From 2020 to 2022, there were a total of 703 *emm*49 cases with most cases occurring in NM (284 cases), followed by MD (228 cases), CA (52 cases), TN (50 cases), CO (43 cases), OR (26 cases), MN (13 cases), GA (5 cases) and CT (2 cases). By rank, *emm*49 increased from the eighth most common *emm* type observed in iGAS ABCs in 2019 to third in 2020 and first in 2021 and 2022.

Along with an increase in *emm*49, the NM ABCs site also saw a spike in an uncommon 11-codon deletion (codons 3–13 of type-specific mature N-terminus) derivative of the *emm*49 *emm* gene called *emm*151. The phylogeny indicates that *emm*151 arose in 4 different instances, with 3 of these instances representing only 1–2 isolates, but with one instance represented by a cluster of 69 isolates primarily recovered from NM. Before 2021, *emm*151 caused only 4 iGAS cases in NM (2 in 2019 and 2 in 2020) and was rare overall, averaging 0.63 cases per ABCs location per year. However, in 2021, a cluster of *emm*151 in New Mexico caused 36 invasive cases, followed by an additional 12 cases in 2022. Together, 50 of the 73 total *emm*151 cases (68.5%) from 2020 through 2022 were found in NM (Fig. S7). The upsurge of *emm*49 and *emm*151 in NM paralleled a rise in both *emm* types in the neighbouring state of Arizona, using samples acquired from statewide genomic surveillance collected between July 2019 and December 2021 [21].

## Discussion

We describe here the recent emergence of *emm*49 as a major cause of invasive disease within multiple states. The first type *emm*49 isolate was first characterized by the CDC Streptococcus Laboratory in 1955 as an M nontypeable isolate, since M types were only serologically characterized up to M47 at the time [22]. Over much of the course of ABCs surveillance, *emm*49 caused a minor fraction (0.18%) of total iGAS in the USA (<5 cases per year). A shift in this pattern was first observed at the turn of the century with a noticeable but temporary rise in cases focused predominantly in CA from 2000 through 2006. In 2015, however, *emm*49 began a rapid expansion among invasive infections that began in the western USA to become a major driver of invasive disease across multiple surveillance sites.

This analysis focused on identifying factors potentially contributing to this rapid upsurge in *emm*49 cases. Epidemiological analysis revealed that the characteristics of persons with *emm*49 invasive cases differed in key ways compared to persons infected with other *emm* types. When compared with non-*emm*49 cases, a higher proportion of the individuals with *emm*49 invasive disease were males, 18–64 years of age and had an underlying medical history of either current alcohol abuse, HIV, cirrhosis or chronic kidney disease. They were also more likely to be comprised of PEH and PWID. *S. pyogenes emm*49 is categorized as a generalist in that it shares equal propensity for either throat or skin epithelium [23]. Starting with its initial discovery as a nephritogenic strain, *emm*49 has been disproportionately associated with skin infections, which are more common among PEH experiencing crowded and less sanitary living conditions [22,23].

Phylogenetic analysis established that *emm*49 invasive isolates collected through ABCs consisted of three major sublineages. Time-scaled phylogenetic analysis found that the tMRCA of all three clades was approximately 1981, while the tMRCA for the post-2015 expansion isolates (clades B and C) was approximately 2004. The phylogeographic analysis also found that there was a considerable amount of time that passed between clade A and the ancestor that would give rise to clades B and C and that this ancestral strain did not evolve from clade A but was introduced separately later. It follows that these ancestral strains were circulating for many years before they were captured as invasive cases in ABCs. Potentially, the ancestral lineages were either circulating inside the ABCs catchment areas as non-invasive strains or outside of the ABCs areas (either nationally or internationally) during that time. It is possible that the current *emm*49 outbreak (clades B and C) emerged through separate introductions of *emm*49 into PEH and/or PWID subpopulations.

Identification of clusters of invasive GAS infections among these high-risk groups is not unique to the *emm49* lineage. Previous work from our group quantified cluster propensity using odds ratios and found that (1) *emm* types vary considerably in their tendency to cluster and (2) *emm* types that are more likely to cluster also have a higher proportion of cases from PEH or PWID [6]. The *emm*49 lineage was the most extreme example of this phenomenon that we observed within ABCs. Using the cluster odds ratios, we showed that *emm*49 had a substantially higher propensity to cluster than any other *emm* type in our collection. We have previously proposed that *emm* types with a high clustering propensity may not spread well in the general population but, given the lack of immune exposure and crowded living conditions, may have an advantage when spreading as skin infections within disadvantaged communities such as PEH and PWID [6].

iGAS incidence has nearly doubled during the past decade, with this increase coinciding with a disproportionate increase rate of disease among PEH or PWID [9]. It is striking that the primary strains afflicting PEH and PWID are *emm* types 49, 82, 92, 77 and 59. All of these *emm* types have been described as tissue generalists and members of *emm* region pattern E or M cluster E (classification systems based on the arrangement of emm and emm-like genes or phylogenetic clustering of M proteins, respectively) that have equal propensity for impetigo or throat reservoirs [9,24, 25]. Within ABCs, the ten *emm* types (including the aforementioned five types) with the highest proportions of cases from the disadvantaged (i.e. PEH and PWID) are all pattern E and D *emm* types and display the highest cluster odds ratios [6]. During this time, iGAS strain types have markedly shifted, with increasing association with cellulitis (https://www.cdc.gov/abcs/bact-facts/data-dashboard.html). Not coincidentally, temporally related patterns E and D *emm* types are the primary drivers of the dramatically increased incidence of iGAS strains with combined resistance to macrolides and clindamycin [19]. These observations are consistent with the notion that increasing economic and social marginalization and social determinants of health may be important mediators of the changing iGAS strain epidemiology within the USA. Looking towards future studies, it may also be beneficial to track the mode of transmission (throat or skin) and employ bacterial GWAS approaches to identify genomic determinants associated with the emergence of these kinds of *emm* types within at-risk populations.

Because our surveillance only tracks invasive disease, one limitation of this study is that we do not know how carriage and non-invasive disease are impacting *emm*49 transmission. Previous work has shown that for several *emm* types, pharyngitis and invasive isolates belonged to the same genomic cluster, suggesting a common connection between these populations [26]. We do not know if a similar phenomenon is happening here. Another limitation is that, given the localized nature and speed at which this expansion and decline can occur, it is likely that we are missing a substantial amount of *emm*49 invasive clustering occurring outside of ABCs areas.

We used a genomic epidemiological approach to describe the recent post-2015 emergence of *emm*49 iGAS in the USA. We find that *emm*49 invasive disease is associated with several patient attributes, including age, residency status and injection drug use. Phylogenetic analysis indicates that the lineage driving the increase was a recent introduction first emerging in the western USA. This work identifies patient, microbial and phylogenetic features associated with the rapid emergence of *emm*49 as a major cause of iGAS across multiple U.S. states.

## Supplementary material

10.1099/mgen.0.001615 Uncited Supplementary Material 1.

10.1099/mgen.0.001615 Uncited Table S1.

## Abbreviations

ABCs: Active Bacterial Core surveillance
CA: California
CDC: Centers for Disease Control and Prevention
CLI: clindamycin
CO: Colorado
CT: Connecticut
ERY: erythromycin
GA: Georgia
GAS: group A *Streptococcus*
HPD: highest posterior density
iGAS: invasive group A *Streptococcus*
MD: Maryland
MN: Minnesota
MRCA: most recent common ancestor
NM: New Mexico
NY: New York
OR: Oregon
PEH: people experiencing homelessness
PWID: people who inject drugs
tMRCA: time to most recent common ancestor
TN: Tennessee
WGS: whole-genome sequencing

## References

[1] Walker MJ, Barnett TC, McArthur JD, Cole JN, Gillen CM (2014). Disease manifestations and pathogenic mechanisms of group A *Streptococcus*. Clin Microbiol Rev.

[2] Beall B, Facklam R, Thompson T (1996). Sequencing emm-specific PCR products for routine and accurate typing of group A streptococci. J Clin Microbiol.

[3] Facklam RF, Martin DR, Lovgren M, Johnson DR, Efstratiou A (2002). Extension of the Lancefield classification for group A streptococci by addition of 22 new M protein gene sequence types from clinical isolates: emm103 to emm124. Clin Infect Dis.

[4] Nelson GE, Pondo T, Toews K-A, Farley MM, Lindegren ML (2016). Epidemiology of invasive group A streptococcal infections in the United States, 2005-2012. Clin Infect Dis.

[5] Metzgar D, McDonough EA, Hansen CJ, Blaesing CR, Baynes D (2010). Local changes in rates of group A *Streptococcus* disease and antibiotic resistance are associated with geographically widespread strain turnover events. Virulence.

[6] Metcalf B, Nanduri S, Chochua S, Li Y, Fleming-Dutra K (2022). Cluster transmission drives invasive group A streptococcus disease within the United States and is focused on communities experiencing disadvantage. J Infect Dis.

[7] Gregory CJ, Okaro JO, Reingold A, Chai S, Herlihy R (2025). Invasive group A streptococcal infections in 10 US states. JAMA.

[8] O’Brien KL, Beall B, Barrett NL, Cieslak PR, Reingold A (2002). Epidemiology of invasive group A *Streptococcus* disease in the United States, 1995-1999. Clin Infect Dis.

[9] Valenciano SJ, Onukwube J, Spiller MW, Thomas A, Como-Sabetti K (2021). Invasive group A streptococcal infections among people who inject drugs and people experiencing homelessness in the United States, 2010–2017. Clin Infect Dis.

[10] Chochua S, Metcalf BJ, Li Z, Rivers J, Mathis S (2017). Population and whole genome sequence based characterization of invasive group A streptococci recovered in the United States during 2015. mBio.

[11] Nanduri SA, Metcalf BJ, Arwady MA, Edens C, Lavin MA (2019). Prolonged and large outbreak of invasive group A *Streptococcus* disease within a nursing home: repeated intrafacility transmission of a single strain. Clin Microbiol Infect.

[12] Gardner SN, Slezak T, Hall BG (2015). kSNP3.0: SNP detection and phylogenetic analysis of genomes without genome alignment or reference genome. Bioinformatics.

[13] Letunic I, Bork P (2016). Interactive tree of life (iTOL) v3: an online tool for the display and annotation of phylogenetic and other trees. Nucleic Acids Res.

[14] Rambaut A, Lam TT, Max Carvalho L, Pybus OG (2016). Exploring the temporal structure of heterochronous sequences using TempEst (formerly Path-O-Gen). Virus Evol.

[15] Bouckaert R, Vaughan TG, Barido-Sottani J, Duchêne S, Fourment M (2019). BEAST 2.5: an advanced software platform for Bayesian evolutionary analysis. PLoS Comput Biol.

[16] Featherstone LA, Rambaut A, Duchene S, Wirth W (2024). Clockor2: inferring global and local strict molecular clocks using root-to-tip regression. Syst Biol.

[17] Rambaut A, Drummond AJ, Xie D, Baele G, Suchard MA (2018). Posterior summarization in Bayesian phylogenetics using tracer 1.7. Syst Biol.

[18] Bielejec F, Baele G, Vrancken B, Suchard MA, Rambaut A (2016). SpreaD3: interactive visualization of spatiotemporal history and trait evolutionary processes. Mol Biol Evol.

[19] Li Y, Rivers J, Mathis S, Li Z, McGee L (2023). Continued increase of erythromycin nonsusceptibility and clindamycin nonsusceptibility among invasive group A streptococci driven by genomic clusters, United States, 2018-2019. Clin Infect Dis.

[20] Prasad N, Rhodes J, Deng L, McCarthy NL, Moline HL (2023). Changes in the incidence of invasive bacterial disease during the COVID-19 pandemic in the United States, 2014-2020. J Infect Dis.

[21] Yaglom HD, Bhattarai R, Lemmer D, Rust L, Ridenour C (2024). Large clusters of invasive emm49 group A *Streptococcus* identified within Arizona health care facilities through statewide genomic surveillance system, 2019-2021. J Infect Dis.

[22] Updyke EL, Moore MS, Conroy E (1955). Provisional new type of group A streptococci associated with nephritis. Science.

[23] Hall JN, Bah SY, Khalid H, Brailey A, Coleman S (2024). Molecular characterization of *Streptococcus pyogenes* (StrepA) non-invasive isolates during the 2022-2023 UK upsurge. Microb Genom.

[24] Bessen DE, Sotir CM, Readdy TL, Hollingshead SK (1996). Genetic correlates of throat and skin isolates of group A streptococci. J Infect Dis.

[25] Sanderson-Smith M, De Oliveira DMP, Guglielmini J, McMillan DJ, Vu T (2014). A systematic and functional classification of *Streptococcus pyogenes* that serves as a new tool for molecular typing and vaccine development. J Infect Dis.

[26] Li Y, Dominguez S, Nanduri SA, Rivers J, Mathis S (2022). Genomic characterization of group A streptococci causing pharyngitis and invasive disease in Colorado, USA, June 2016– April 2017. J Infect Dis.

