# Supplementary material for: The rise and spread of invasive emm49 Streptococcus pyogenes in the USA
Source: Microb Genom. 2026 Jan 29;12(1):001615. doi: 10.1099/mgen.0.001615 (PMC12856021; doi:10.1099/mgen.0.001615)
Supplement: Uncited Supplementary Material 1. [file mgen-12-01615-s001.pdf]

Supplement

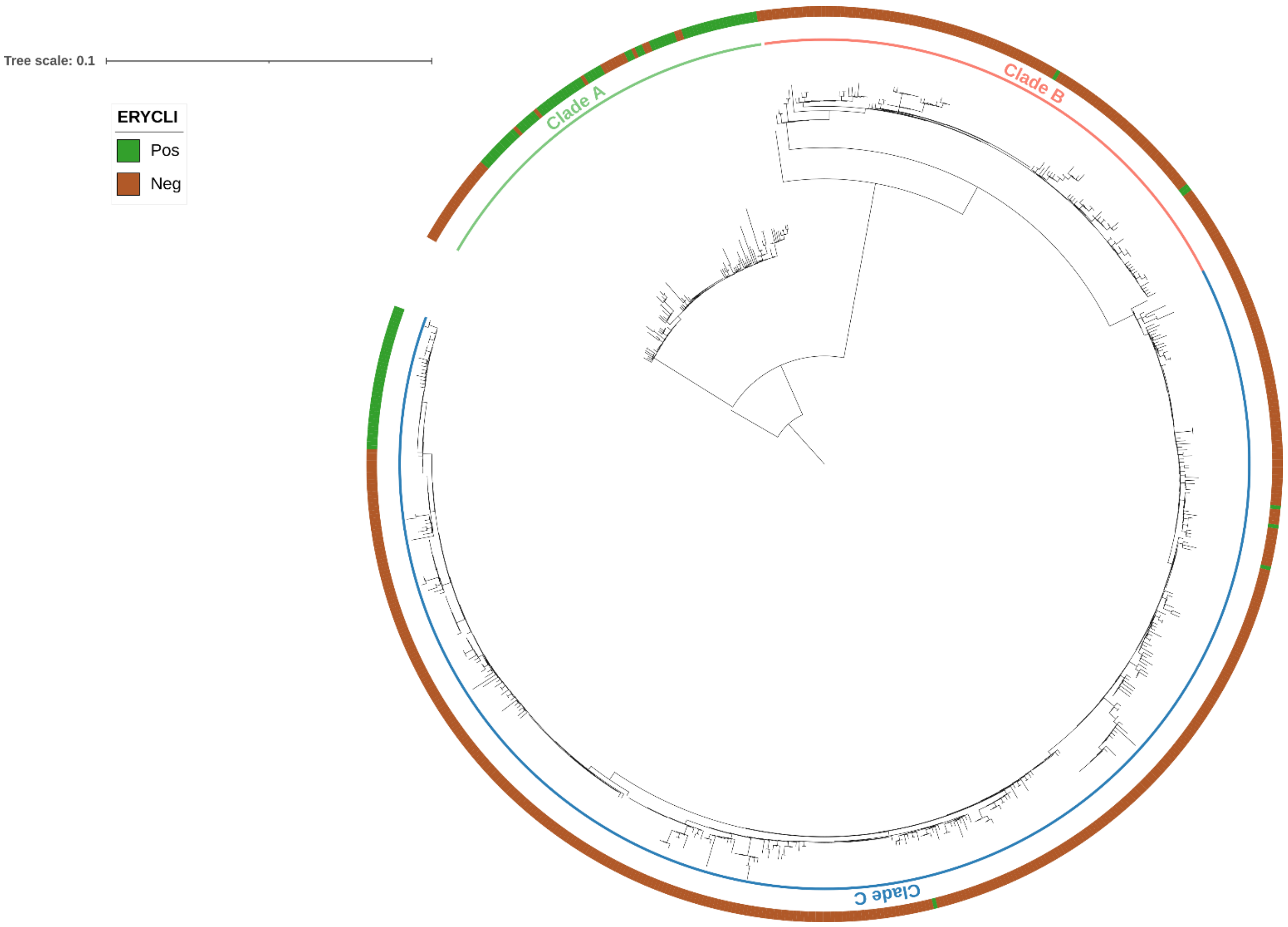

Figure S1: A midpoint rooted phylogeny representing 726 *emm*49 isolates constructed from an alignment of 782 core SNPs. Combined Erythromycin/clindamycin resistance is uncommon but highly clonally associated among invasive *emm*49 strains. Invasive *emm*49 isolates were, for the most part, erythromycin and clindamycin sensitive. However, 68 of the 106 isolates of clade A (64%) and 38 isolates from a subclade of clade C (8% of the 471 clade C isolates) were both highly enriched for erythromycin/clindamycin non-susceptibility. Outside of the clonal emergence in clade A and a clade C subclade, erythromycin and clindamycin appeared sporadically in 3 isolates of clade B and 4 additional isolates of clade C.

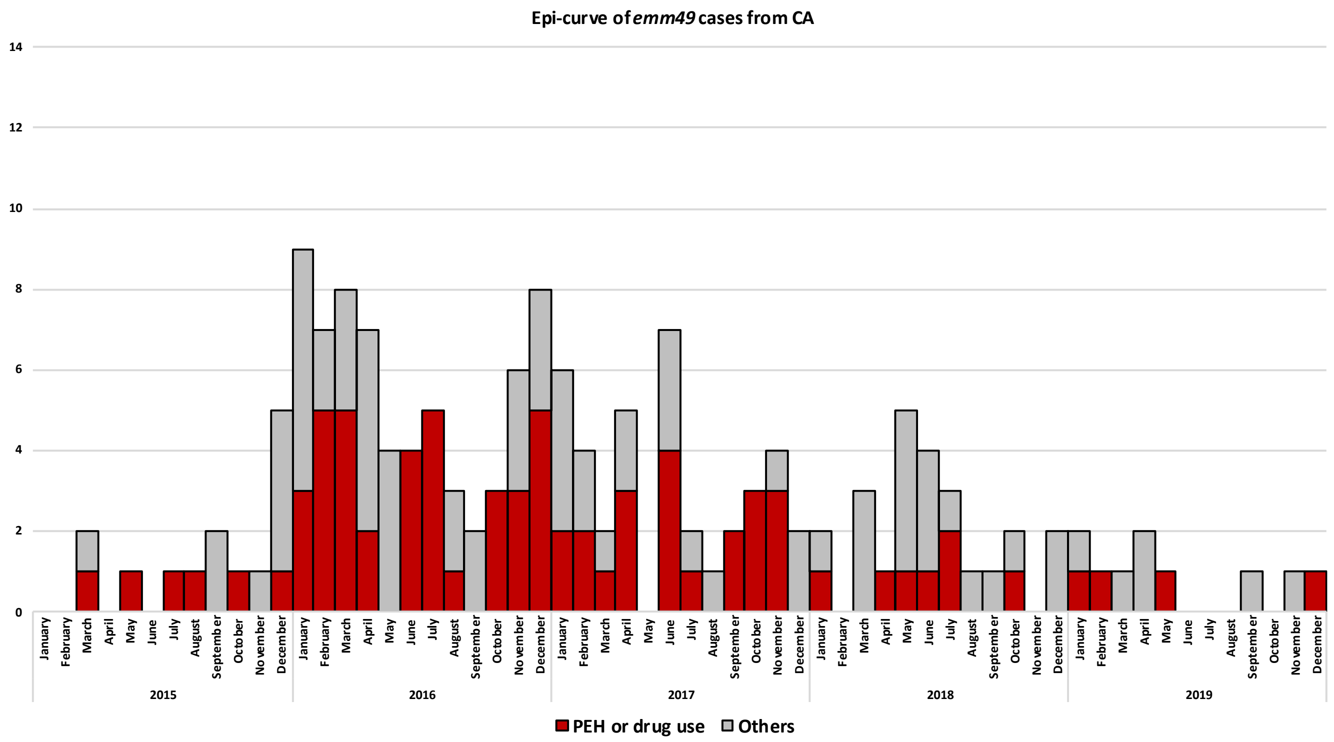

Figure S2: *emm*49 invasive GAS cases reported from California in Active Bacterial Core surveillance, 2015-2019.

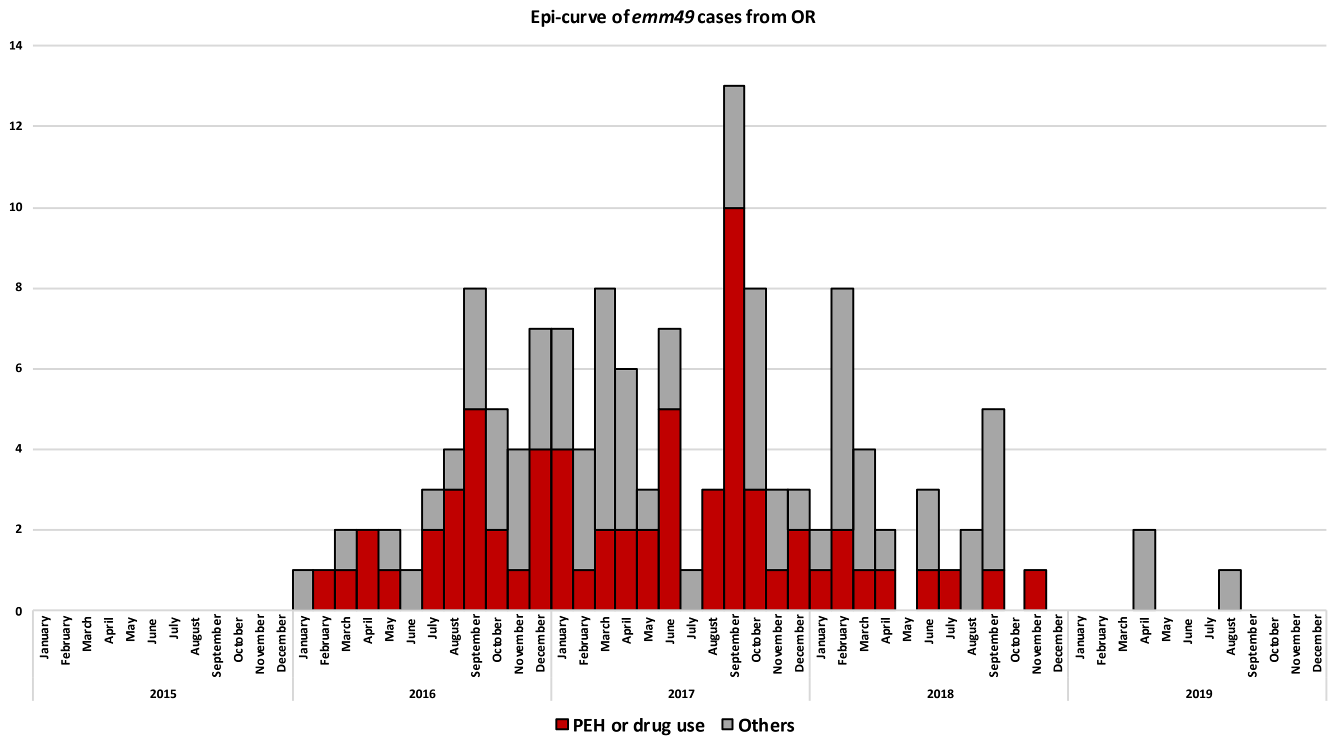

Figure S3: *emm*49 invasive GAS cases reported from Oregon in Active Bacterial Core surveillance, 2015-2019.

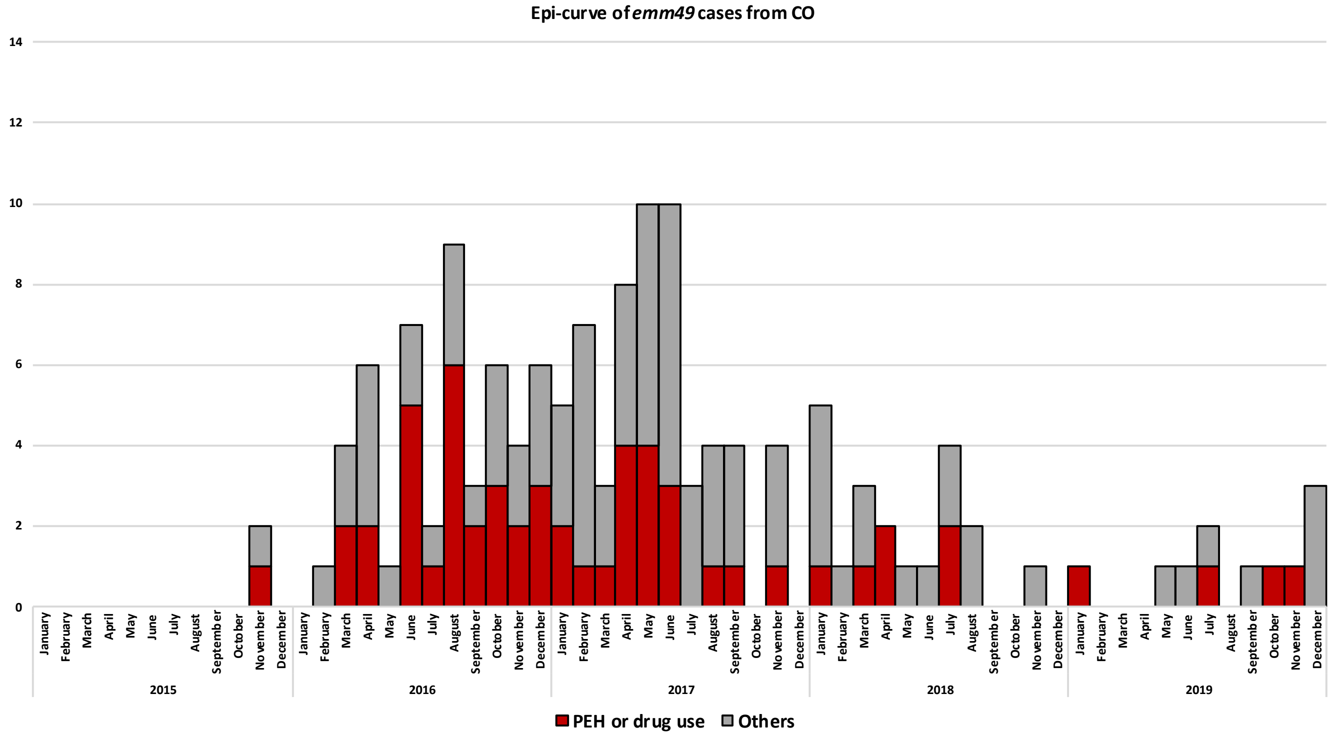

Figure S4: *emm*49 invasive GAS cases reported from Colorado in Active Bacterial Core surveillance, 2015-2019.

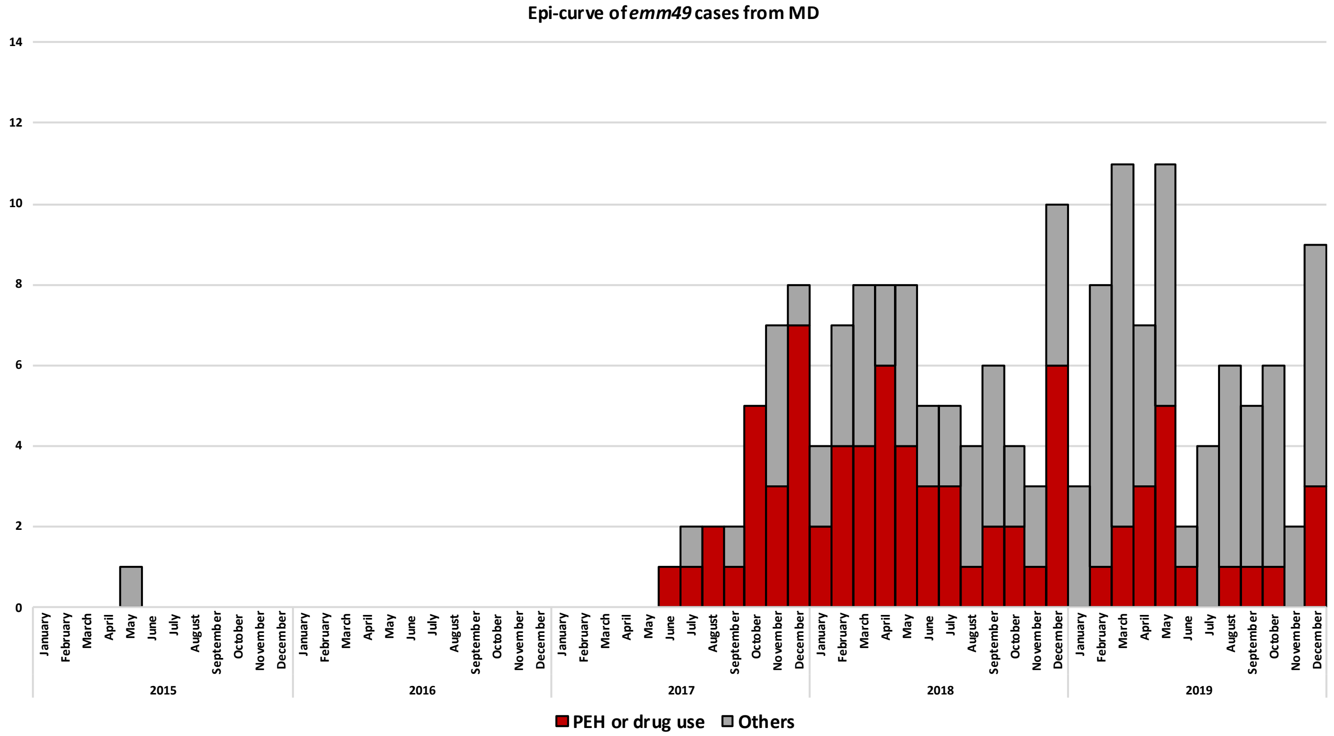

Figure S5: *emm*49 invasive GAS cases reported from Maryland in Active Bacterial Core surveillance, 2015-2019.

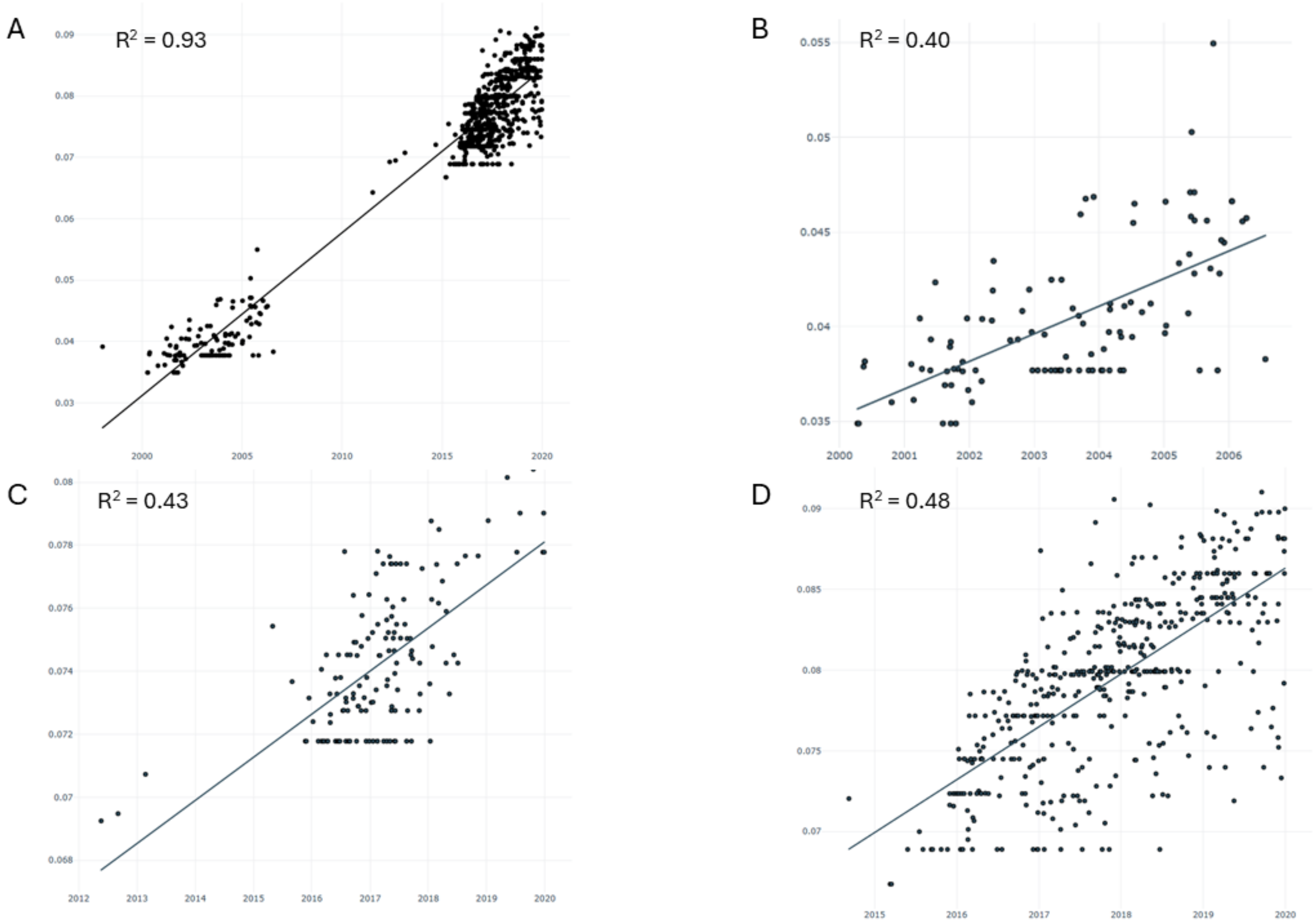

Figure S6: Root-to-tip regression plots showing the strength of the molecular clock signal for the full collection of 726 emm49 genomes as well as individual subclades. A) The Root-to-tip regression of the full *emm*49 dataset representing 726 genomes spanning 1998 – 2020 yields a coefficient of determination (R^2) of 0.93. B) Root-to-tip regression of clade A representing 106 genomes from 2000 – 2006 generates a R^2 of 0.40. C) Root-to-tip regression of clade B representing 147 genomes from 2012 – 2020 yields a R^2 of 0.43. D) Root-to-tip regression of clade C representing the largest subclade of 471 genomes spanning 2014 – 2020 outputs a R^2 of 0.48.

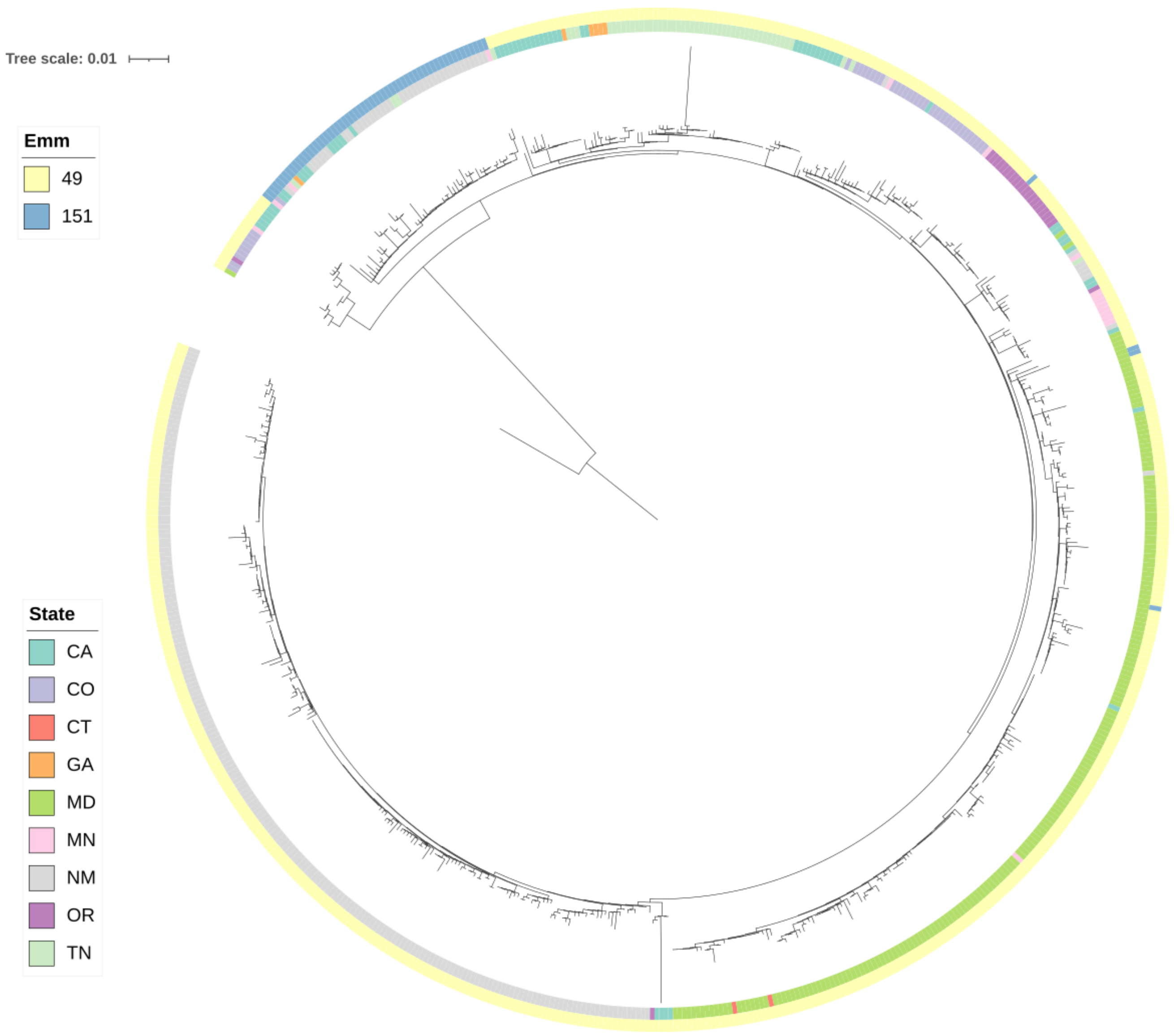

Figure S7: A midpoint rooted phylogeny representing 658 *emm*49 isolates constructed from an alignment of 1186 core SNPs. *emm*49 (and its genetic derivative *emm*151) continued to be a major cause of invasive GAS post 2020. From 2020-2022, *emm*49 invasive disease cases were observed in all ABC surveillance sites except New York. Most cases occurred in New Mexico (284 cases) and Maryland (228 cases) but were also observed in California (52 cases), Tennessee (50 cases), Colorado (43 cases), Oregon (26 cases), Minnesota (13 cases), Georgia (5 cases), and Connecticut (2 cases). A genetic variant of *emm*49, called *emm*151, was responsible for a cluster of iGAS primarily centered within New Mexico (40 cases) and California (10 cases) but also observed sporadically in Colorado, Georgia, Maryland, Minnesota, Oregon, and Tennessee (1 to 3 cases each).
